# The role of Seladelpar in primary biliary cholangitis: a systematic review and meta-analysis

**DOI:** 10.1186/s12876-025-03812-3

**Published:** 2025-04-05

**Authors:** Taimoor Ashraf, Omar Abunada, Nandlal Seerani, Kashif Ali, Areej Muhammad, Syeda Lamiya Mir, Syed Adil Mir Shah, Muhammad Hassaan, Vikash Kumar, Waseem Abbas, Simran Bajaj, Asfia Qammar, F. N. U. Deepak, Salih Abdella Yusuf

**Affiliations:** 1 Nishtar Medical University, Multan, Pakistan; 2 Liaquat University of Medical and Health Sciences, Jamshoro, Karachi, Pakistan; 3 University of Texas Rio Grande Valley, Edinburg, USA; 4 Dow Medical College, Dow University of Health Sciences, Karachi, Pakistan; 5 Jinnah Sindh Medical University, Karachi, Pakistan; 6 Shaheed Mohtarma Benazir Bhutto Medical University, Larkana, Pakistan; 7 Shaheed Mohtarma Benazir Bhutto Medical College Lyari, Karachi, Pakistan; 8 Hawassa University, Hawassa, Ethiopia

**Keywords:** Primary biliary cholangitis, Seladelpar, Liver function, PPAR-δ, Meta-analysis

## Abstract

**Introduction:**

Primary biliary cholangitis (PBC) is a chronic autoimmune liver disease characterized by progressive bile duct destruction, leading to cholestasis and, if untreated, liver failure. Although ursodeoxycholic acid (UDCA) remains the first-line treatment, many patients exhibit an inadequate response, necessitating alternative therapeutic options. Seladelpar, a peroxisome proliferator-activated receptor delta (PPAR-δ) agonist, has emerged as a promising alternative due to its anti-inflammatory and anti-fibrotic properties.

**Methods:**

A systematic review and meta-analysis of randomized controlled trials (RCTs) were conducted to evaluate the efficacy and safety of Seladelpar in patients with PBC. A comprehensive database search was performed to identify studies comparing Seladelpar with placebo. Primary and secondary outcomes, including alkaline phosphatase (ALP) normalization, biochemical response, and adverse events, were analyzed.

**Results:**

Three RCTs, comprising 496 patients, were included. Seladelpar significantly improved ALP normalization and biochemical response compared to placebo. Additionally, it effectively reduced ALP and ALT levels from baseline to follow-up. Adverse events, including abdominal pain and headache, were reported, with a higher incidence observed in the Seladelpar group, while other adverse events showed no significant differences between groups.

**Conclusion:**

Seladelpar appears to be an effective treatment for PBC, demonstrating significant improvements in key liver function markers. While it has shown therapeutic benefits, further research is warranted to evaluate its long-term safety, particularly regarding adverse event incidence, and to determine its efficacy across different dosages.

**Supplementary Information:**

The online version contains supplementary material available at 10.1186/s12876-025-03812-3.

## Introduction

Primary biliary cholangitis (PBC) is a chronic autoimmune liver disease characterized by the progressive destruction of intrahepatic bile ducts, leading to cholestasis, liver fibrosis, and, if untreated, potential liver failure [1–3]. In recent years, the prevalence of PBC has been rising, with the highest incidence observed in Europe and North America. The disease predominantly affects middle-aged women, accounting for over 90% of cases, highlighting the urgent need for effective therapeutic interventions to mitigate its clinical impact [3, 4].

Although ursodeoxycholic acid (UDCA) remains the primary treatment for PBC, a significant proportion of patients exhibit an inadequate response or intolerance to this standard therapy, underscoring the necessity for alternative treatment options [5]. Seladelpar, a selective peroxisome proliferator-activated receptor delta (PPAR-δ) agonist, has emerged as a promising therapeutic agent for PBC management [6]. PPAR-δ activation plays a crucial role in modulating inflammatory and fibrotic processes in liver diseases, making Seladelpar an attractive candidate for PBC treatment [7].

Seladelpar has demonstrated significant improvements in biochemical markers of cholestasis, liver function tests, and histological features, while also addressing safety concerns and alleviating symptoms such as pruritus, sleep disturbances, fatigue, and elevated serum bile acid levels in affected patients [8, 9]. However, its use has been associated with a decrease in low-density lipoprotein (LDL) levels and the need for enhanced blood pressure monitoring, though data on these effects remain limited [10].

Despite promising findings from preliminary studies, the safety and efficacy of Seladelpar in PBC treatment remain incompletely understood, with existing literature presenting conflicting results regarding its clinical benefits. Concerns persist about its long-term safety profile, particularly its potential impact on hepatological outcomes and other adverse effects.

To address these knowledge gaps, we conducted a systematic review and meta-analysis of published literature to comprehensively evaluate the safety and efficacy of Seladelpar in PBC treatment. By synthesizing data from relevant clinical trials, our study aims to provide evidence-based insights into Seladelpar’s therapeutic potential. Additionally, we seek to identify areas for future research and development, ultimately striving to improve outcomes for patients suffering from this debilitating liver disease.

## Methods

### Data sources and search strategy

This meta-analysis was conducted in accordance with the Preferred Reporting Items for Systematic Reviews and Meta-Analyses (PRISMA) guidelines [11] and has been registered in PROSPERO under the ID CRD420250652058. A comprehensive literature search was performed across multiple databases, including PubMed, Scopus, Cochrane Library, ScienceDirect, and Google Scholar, covering studies from inception until March 2025. The detailed search strategy employed for each database is provided in Supplementary Table 1. To ensure a thorough review, the bibliographies of all retrieved articles were screened for additional relevant studies. Furthermore, grey literature sources were explored, including clinical trial registries such as ClinicalTrials.gov, regulatory reports from the U.S. Food and Drug Administration (FDA), and conference proceedings for relevant abstracts and presentations. Preprint servers were also searched for dissertations and theses. However, challenges associated with grey literature included the lack of standardized indexing, limited methodological details, variability in reporting formats, and the absence of peer review in certain sources, making the assessment of study quality and relevance more complex.

### Study selection and eligibility criteria

A total of 798 articles retrieved from the systematic search were imported into the EndNote reference library (version X8.1, Clarivate Analytics), where duplicate records were removed. Two independent investigators conducted an initial screening at the title and abstract level, followed by a full-text review to confirm eligibility. Any disagreements were resolved through discussion, with a third investigator consulted if needed. Studies were included if they met the following pre-specified eligibility criteria: (I) published randomized controlled trials (RCTs) with no date restrictions, (II) studies comparing outcomes in patients receiving Seladelpar versus placebo, and (III) studies reporting at least one outcome of interest. All other study types, including case series, observational studies, and non-randomized trials, were excluded.

### Data extraction and quality assessment

Data extracted from the included studies encompassed patient baseline characteristics and key outcomes, including normalization of alkaline phosphatase (ALP) and biochemical response. The biochemical response was defined by Hirschfield et al. [12] as ALP < 1.67 × upper limit of normal (ULN), ≥ 15% ALP decrease from baseline, and total bilirubin ≤ ULN at month 3, while Hirschfield et al. [13] defined it as ALP < 1.67 × ULN, ≥ 15% ALP decrease from baseline, and normal total bilirubin at month 12. Additional outcomes included changes in ALP, alanine aminotransferase (ALT), and total serum bilirubin from baseline, along with adverse events such as pruritus, abdominal pain, headache, and nausea. Two independent reviewers assessed the quality of the included RCTs using the Cochrane Risk of Bias Tool for randomized controlled trials (ROB-2) [14]. Any discrepancies in quality assessment were resolved through discussion, with a third investigator providing adjudication when necessary.

### Statistical analysis

All statistical analyses were performed using Review Manager (RevMan, version 5.4.1; Copenhagen: The Nordic Cochrane Centre, The Cochrane Collaboration, 2014). Continuous and dichotomous outcomes were analyzed using standardized mean differences (SMD) for continuous variables and risk ratios (RR) with 95% confidence intervals (CI) for dichotomous variables. Results were synthesized using a random-effects model to account for potential variability across the included studies. This model assumes that differences between study estimates stem from both within-study and between-study variation, making it particularly suitable for meta-analyses with clinical and methodological heterogeneity. Between-study variance (τ^2^) was estimated using the DerSimonian and Laird method. Heterogeneity was assessed using the I^2^ statistic, with thresholds of 25%, 50%, and 75% representing low, moderate, and high heterogeneity, respectively. If heterogeneity exceeded 75%, a leave-one-out sensitivity analysis was performed to assess the influence of individual studies on the overall results [15]. Additionally, subgroup analyses were conducted to examine potential dose–response relationships across different Seladelpar dosages (5 mg, 10 mg, 50 mg, and 200 mg). Statistical significance was set at *p* ≤ 0.05.

## Results

### Study selection and characteristics

**Fig. 1:**
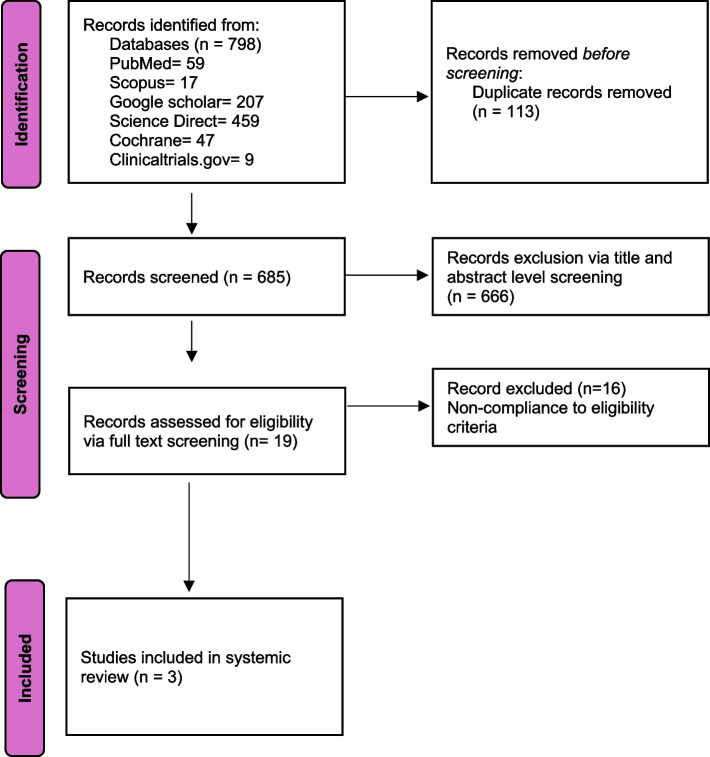
PRISMA flow chart

**Fig. 2:**
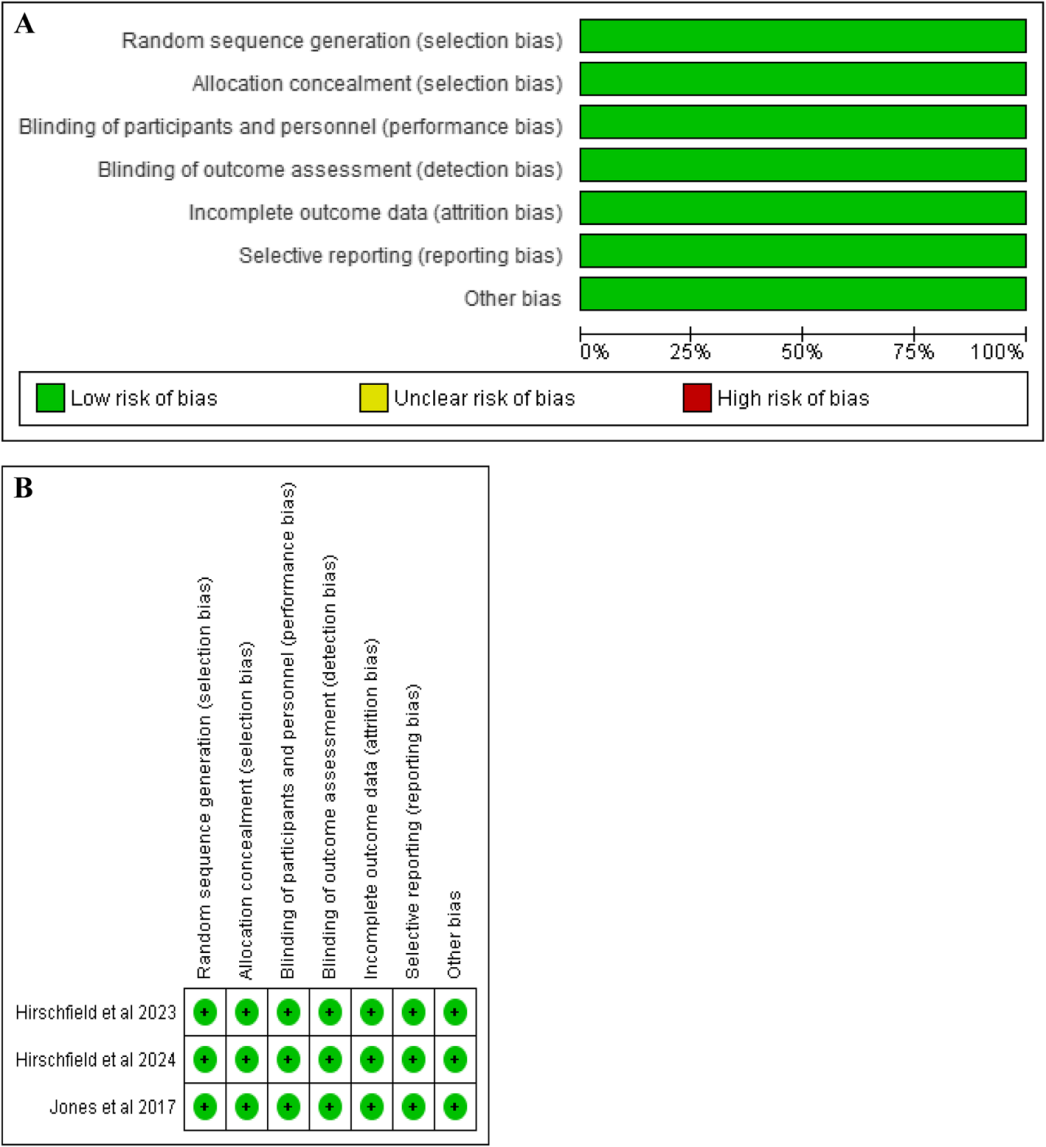
**A** Risk of Bias Graph. **B.** Risk of Bias Summary

A comprehensive literature search yielded 798 articles. After removing duplicates and ineligible studies, three RCTs were included in this meta-analysis. The PRISMA flowchart summarizes the study selection process (Fig. 1). These three studies [6, 12, 13] included 496 patients, with 331 in the Seladelpar group and 165 in the placebo group. The follow-up duration ranged from 6 to 12 months. The mean age of patients was 55.91 years in the Seladelpar group and 55.97 years in the placebo arm. The general study characteristics and baseline characteristics of the included studies are summarized in Tables 1 and 2. The risk of bias assessment details are presented in Fig. 2A, B, and Supplementary Table 2, with all included studies rated as high quality.

**Table 1:** General characteristics of included studies table

Study ID	Clinical trial no	Study Design	Study Duration	Country	Dose of Seladelpar	Sample Size (n)		Follow up Visit	Outcomes Measure
						**Seladelpar**	**Placebo**		
Jones et al. 2017 [6]	NCT02609048	Multicentre, double-blind, randomised, placebo-controlled, parallel, dose-ranging trial	Nov 4, 2015, and May 26, 2016 (18 weeks)	29 sites in North America and Europe	50 mg	13	13	2 weeks	Primary outcome: Change in alkaline phosphatase levels over 12 weeks. Secondary outcomes encompass safety, tolerability, liver enzyme levels (AST, ALT, GGT), lipid profile, pruritus assessment, and exploratory measures including bile acid levels, inflammatory markers, and trough plasma concentrations of seladelpar and its metabolites at weeks 4 and 12
					200 mg	12			
Hirschfield et al. 2023 [12]	NCT03602560	Phase 3, double-blind, randomized, placebo-controlled study	November 26, 2018, and November 12, 2019	111 sites in 21 countries	5 mg orally	89	87	4 weeks	Primary outcomes: Composite biochemical response, ALP change from baseline, and total bilirubin after month 3. secondary endpoints: ALP normalization and change in pruritus NRS from baseline at month 3
					10 mg orally	89			
Hirschfield et al. 2024 [13]	NCT04620733	Phase 3, multicenter, double-blind, randomized, placebo-controlled trial	Up to 12 months	90 sites in 24 countries	10 mg daily	128	65	2 weeks	Primary outcomes; biochemical response and total billirubin levels at 12 months. Secondary outcomes: Normalization of alkaline phosphatase level and change in pruritis

*NCT* National Clinical Trial, *mg* milligram

**Table 2:** Patient baseline characteristics table

Study ID	Male/Female		Age (Mean, SD)		BMI (kg/m2)		ALP Level U/L (Mean, SD)		Total bilirubin level/ mg/Dl (Mean, SD)		Pruritis NRS score (Mean, SD)	
	Seladelpar	Placebo	Seladelpar	Placebo	Seladelpar	Placebo	Seladelpar	Placebo	Seladelpar	Placebo	Seladelpar	Placebo
Jones et al. 2017 [6]	1:12	1:12	54 ± 7.47	55 ± 10.79	24 ± 5	28 ± 6	312 ± 95	233 ± 73	0·73 ± 0·27	0·68 ± 0·35	-	-
	0:12		58.67 ± 9.22		27 ± 4		248 ± 89		0·75 ± 0·38		-	
Hirschfield et al. 2023 [12]	-	-	54.7 ± 9.7	55.9 ± 8.2	27.7 ± 6.1	28.2 ± 5.5	290.5 ± 104.2	293.4 ± 106.2	0.76 ± 0.35	0.71 ± 0.32	2.8 ± 2.5	2.9 ± 2.5
			55.6 ± 9.1		27.6 ± 5.9		290.8 ± 109.1		0.72 ± 0.32		2.7 ± 2.6	
Hirschfield et al. 2024 [13]	-	-	56.6 ± 10.0	57.0 ± 9.2	-	-	314.6 ± 123.0	313.8 ± 117.7	0.77 ± 0.3	0.74 ± 0.3	3.0 ± 2.8	3.0 ± 3.0

*ALT* Alkaline phosphatase, *SD* Standard deviation

### Primary outcome

#### Normalization of ALP

**Fig. 3:**
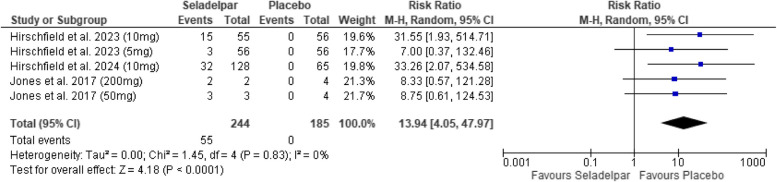
Forest plot for normalization of ALP

A meta-analysis of all studies demonstrated that Seladelpar significantly increased ALP normalization compared to placebo (RR: 13.94, 95% CI [4.05, 47.97]; *p* < 0.0001, I^2^ = 0%), as shown in Fig. 3.

### Secondary outcomes

I. Biochemical Response
  A random-effects meta-analysis of all studies showed that Seladelpar was associated with a significantly greater biochemical response compared to placebo (RR: 4.18, 95% CI [2.74, 6.37]; *p* < 0.00001, I^2^ = 25%), as illustrated in Supplementary Figure 1.
II. ALP Change from Baseline to Longest Follow-up
  All studies assessed changes in ALP levels from baseline to follow-up, revealing that Seladelpar significantly reduced ALP levels compared to placebo (SMD: −2.06, 95% CI [−2.87, −1.25]; *p* < 0.00001, I^2^ = 84%), as shown in Supplementary Figure 2A.

### Assessment of heterogeneity

To explore the source of high heterogeneity in ALP change, we conducted a sensitivity analysis by excluding the outlier study, Jones et al. [6]. After exclusion, I^2^ dropped from 84 to 0%, and the results remained significant (SMD: −1.32, 95% CI [−1.59, −1.05]; *p* < 0.00001, I^2^ = 0%), as shown in Supplementary Figure 2B.

III. ALT Change from Baseline to Longest Follow-up
  All studies assessed changes in ALT levels. The pooled analysis showed that Seladelpar significantly lowered ALT levels compared to placebo (SMD: −0.55, 95% CI [−0.80, −0.30]; *p* < 0.0001, I^2^ = 0%), as illustrated in Supplementary Figure 3.
IV. Adverse Events
  All studies reported adverse and serious adverse events. The pooled results showed that there was no significant difference in the incidence of adverse events between Seladelpar (225/306) and placebo (183/239) (RR: 0.94, 95% CI [0.83, 1.07]; *p* = 0.33, I^2^ = 40%). Similarly, there was no significant difference in serious adverse events between Seladelpar (13/306) and placebo (10/239) (RR: 0.91, 95% CI [0.39, 2.14]; *p* = 0.83, I^2^ = 0%), as shown in Supplementary Figure 4.
V. Pruritus
  Pruritus was reported in all studies. The pooled analysis indicated that 7.3% of patients in the Seladelpar group (24/331) and 12.8% in the placebo group (34/265) experienced pruritus, with no significant difference between groups (RR: 0.62, 95% CI [0.27, 1.40]; *p* = 0.25, I^2^ = 49%), as shown in Supplementary Figure 5.
VI. Abdominal Pain
  The pooled analysis revealed that 7.9% of patients in the Seladelpar group (26/331) and 2.6% in the placebo group (7/265) reported abdominal pain, with a significantly higher incidence in the Seladelpar group (RR: 2.73, 95% CI [1.24, 6.02]; *p* = 0.01, I^2^ = 0%), as shown in Supplementary Figure 6.
VII. Headache
  Headache was reported across all studies. The pooled analysis showed that 7.2% of patients in the Seladelpar group (22/306) and 1.7% in the placebo group (4/239) experienced headaches, with a significantly higher incidence in the Seladelpar group (RR: 3.84, 95% CI [1.34, 11.00]; *p* = 0.01, I^2^ = 0%), as shown in Supplementary Figure 7.
VIII. Nausea
  Nausea was reported in all studies, with 7.3% of patients in the Seladelpar group (24/331) and 4.9% in the placebo group (13/265) experiencing nausea. However, the difference was not statistically significant (RR: 1.51, 95% CI [0.78, 2.93]; *p* = 0.22, I^2^ = 0%), as shown in Supplementary Figure 8.
IX. Total Serum Bilirubin
  The pooled analysis showed no significant difference in total serum bilirubin levels between the Seladelpar and placebo groups (SMD: −0.15, 95% CI [−0.32, 0.02]; *p* = 0.08, I^2^ = 0%), as shown in Supplementary Figure 9.
X. Any adverse event leading to treatment discontinuation
  The pooled analysis indicated that 3.9% of patients in the Seladelpar group (13/331) and 2.6% in the placebo group (7/265) discontinued treatment due to adverse events, with no statistically significant difference between groups (RR: 1.18, 95% CI [0.48, 2.92]; *p* = 0.72, I^2^ = 0%), as shown in Supplementary Figure 10.

### Subgroup analysis

A subgroup analysis was conducted based on dosage to elucidate potential dose–response relationships. Subgroups were established for outcomes wherever feasible, facilitating a more granular evaluation.

### Normalization of ALP

No statistically significant differences were observed among the subgroups receiving 5 mg (RR: 7.00 [0.37, 132.46]; *p* = 0.19), 50 mg (RR: 8.75 [0.61, 124.53]; *p* = 0.11), and 200 mg (RR: 8.33 [0.57, 121.28]; *p* = 0.12). However, the 10 mg subgroup exhibited a statistically significant effect (RR: 32.40 [4.52, 232.08]; *p* = 0.0005; I^2^ = 0%), indicating a greater proportion of patients achieving ALP normalization in the Seladelpar-treated cohort (Supplementary Figure 11).

### Biochemical response

A statistically significant difference was identified in both the 5 mg subgroup (RR: 4.57 [2.20, 9.48]; *p* < 0.0001) and the 10 mg subgroup (RR: 4.20 [4.18, 6.37]; *p* < 0.0001; I^2^ = 61%) (Supplementary Figure 12).

### ALP change from baseline to longest follow-up

Significant reductions in ALP levels from baseline to the longest follow-up were observed across all dosage subgroups: 5 mg (SMD: −1.10 [−1.71, −0.48]; *p* = 0.0005), 10 mg (SMD: −1.37 [−1.67, −1.07]; *p* < 0.00001; I^2^ = 0%), 50 mg (SMD: −3.29 [−4.52, −2.05]; *p* < 0.00001), and 200 mg (SMD: −4.60 [−6.20, −3.01]; *p* < 0.00001) (Supplementary Figure 13).

### ALT change from baseline to longest follow-up

Statistically significant reductions in ALT levels were also observed in the 5 mg subgroup (SMD: −0.87 [−1.47, −0.27]; *p* = 0.004) and the 10 mg subgroup (SMD: −0.48 [−0.76, −0.21]; *p* = 0.0005; I^2^ = 0%) (Supplementary Figure 14).

### Meta-regression

To investigate potential covariates influencing the effect size on the primary outcome—ALP normalization—meta-regression was performed, incorporating mean age, the proportion of female participants, body mass index (BMI), and disease duration. None of these covariates demonstrated a statistically significant association with ALP normalization, with results as follows: mean age (coefficient: 0.0599, *p* = 0.9410), percentage of female participants (coefficient: 0.0969, *p* = 0.8251), BMI (coefficient: 0.2400, *p* = 0.7838), and disease duration (coefficient: 0.2535, *p* = 0.8071) (Supplementary Figures 15A–15D).

## Discussion

We conducted a meta-analysis to evaluate the safety and efficacy of Seladelpar in patients with PBC, incorporating data from three RCTs [6, 12, 13]. Our findings demonstrated significant improvements in ALP levels and biochemical responses with Seladelpar treatment. However, high heterogeneity was observed in ALP change from baseline to follow-up. Regarding safety, no significant association was found for adverse events and serious adverse events.

Seladelpar, a PPAR-δ agonist, has emerged as a recent addition to the treatment arsenal for PBC for several compelling reasons. Before its introduction, various treatment strategies had been employed, but over time, the efficacy of these drugs waned, or their adverse effects became prohibitive. UDCA, the cornerstone of PBC treatment, is hailed as highly effective. However, in a significant subset of individuals—up to 40% UDCA fails to elicit the desired biochemical response [16]. Additionally, while UDCA remains a mainstay, its efficacy diminishes in some patients over time. Obeticholic acid (OCA), another drug used in PBC management, initially showed promise by reducing enzymatic levels. Unfortunately, its use has been associated with liver damage and the potential for liver failure, limiting its widespread adoption [17, 18]. These challenges underscored the need for novel therapeutic approaches, leading to the introduction of Seladelpar. Additionally, seladelpar offers a solution to these issues by providing a promising alternative. With its unique mechanism of action and potentially favorable safety profile, Seladelpar presents an opportunity to address the limitations of previous treatments and improve outcomes for individuals with PBC [19]. Several studies have underscored Seladelpar’s beneficial effects in improving liver function, reducing enzyme levels, and enhancing cholestatic liver functions [16, 20, 21]. Furthermore, Seladelpar has been shown to act as an anti-inflammatory and anti-fibrotic agent in the liver [22] and has been proposed as a potential alternative in cases where UDCA fails to yield satisfactory results [23]. Seladelpar has also shown promise in relieving pruritus symptoms and enhancing sleep quality [8, 9]. In terms of biochemical response, various studies have reported positive outcomes. For instance, one study reported a reduction in ALP levels consistent with our findings. However, substantial heterogeneity was observed, which decreased to 0% upon performing a leave-one-out analysis excluding Jones et al. This heterogeneity was likely attributable to the lower baseline ALP levels in patients included in Jones et al. compared to the other two studies, which had comparable baseline levels. Additionally, the shorter follow-up duration in Jones et al. (12 weeks) compared to the 12-month follow-up in the remaining studies may have contributed to this variability. Moreover, it also suggested a potential increase in ALT levels, which contradicted our results [6]. Furthermore, because Seladelpar stimulates fatty acid desaturation pathways, the downregulation of CYP7A1 does not lead to unfavorable alterations in lipid profiles, which is a concern with OCA [24].

The effects of different Seladelpar doses on various outcomes are still being investigated. Currently, there is insufficient evidence to suggest that dose adjustments significantly impact the drug’s safety and efficacy. Although one study indicated that lower doses were associated with a reduced incidence of adverse effects and no serious adverse events at the lowest dose [25], this study was prematurely discontinued, warranting further investigation. Recent phase 2 and phase 3 clinical trials have reported no significant safety concerns, reinforcing the drug’s potential tolerability profile [26, 27]. However, concerns regarding the long-term safety of Seladelpar remain, particularly given its intended use for the chronic management of PBC. One study observed that prolonged use over two years did not lead to a cumulative increase in adverse effects, with some patients experiencing a reduction in certain side effects. Nonetheless, at least one patient developed a serious adverse event, necessitating ongoing safety monitoring [25]. This meta-analysis indicates that commonly assessed adverse events, including pruritus, abdominal pain, headache, and nausea, were not significantly different between Seladelpar and placebo, suggesting a favorable safety profile. Beyond these commonly reported adverse effects, it is crucial to evaluate the impact of PPAR-δ agonists on cardiovascular risk, particularly in patients with preexisting dyslipidemia or hypertension, given their established role in lipid metabolism [28]. While some studies have indicated modest reductions in LDL and cholesterol with PPAR-δ, suggesting potential cardiovascular benefits, further long-term data are required to substantiate these findings [29, 30]. Compared to other second-line treatments for PBC, such as OCA, which has been associated with pruritus and hepatotoxicity, or fibrates, which carry a risk of renal impairment and myopathy, the long-term safety profile of Seladelpar requires further elucidation through extended follow-up studies. A comprehensive evaluation of its risk–benefit profile in comparison to existing therapies is essential to determine its suitability for prolonged use.

The potential clinical implications of Seladelpar’s efficacy in improving ALP and ALT levels are multifaceted. Based on the findings of our study, there are various ways through which Seladelpar could be integrated into the management of PBC. It could be used in cases where UDCA fails to show its effect or when patients are intolerant to UDCA. Additionally, it can also be considered when the disease is severe. However, while using this drug, it is important to assess certain parameters for potential side effects. These include lipid profile, blood pressure monitoring, and evaluation for any cardiovascular issues [31]. Similarly, the normalization or significant reduction of these liver enzymes may indicate a decrease in hepatocyte injury and inflammation, leading to the preservation of liver function and potentially delaying the onset of complications such as liver fibrosis and cirrhosis [15, 20]. Moreover, by targeting PPAR-δ, Seladelpar may exert additional beneficial effects on lipid metabolism, insulin sensitivity, and inflammatory pathways, which are dysregulated in PBC and contribute to disease pathogenesis. In terms of patient outcomes, the improvement in ALP and ALT levels with Seladelpar therapy may translate into tangible benefits such as reduced symptoms of fatigue, pruritus (itching), and jaundice, thereby enhancing patients’ quality of life. Additionally, by stabilizing or improving liver function, Seladelpar has the potential to reduce the need for liver transplantation and liver-related hospitalizations, leading to healthcare cost savings and improved healthcare resource utilization [8, 9, 19, 20]. To enhance the assessment of our main result, we conducted a meta-regression analysis to investigate potential relationships between various variables that may impact Seladelpar’s effect on ALP level normalization. Our findings suggested that Seladelpar functions independently of certain variables such as BMI, average age, percentage of female participants, and disease duration. Additionally, due to the inclusion of different doses of our parent drug, we conducted a subgroup analysis, creating different subgroups based on the dosages used. For one outcome, no significant changes were observed when different doses were used. However, the use of 10 mg Seladelpar led to significant benefits. In the biochemical response, participants who used 5 mg showed increased biochemical responses. For the remaining outcomes, higher doses of Seladelpar proved to be beneficial. Overall, while higher doses led to beneficial effects in some endpoints, lower doses had a greater effect in others, indicating that a higher dose is not always superior to a lower dose of the drug. However, an enhanced trial was conducted using different doses of 5, 10, and 25 mg of this drug. The results of that trial showed that the higher the dose, the greater the effect it produced [32]. Similarly, in terms of adverse effects, higher doses posed a greater risk. Higher doses of Seladelpar were associated with an increased risk of gastrointestinal upset, such as nausea and vomiting, along with an increase in fatigue and liver enzyme levels [32].

### Limitations

While our meta-analysis provides valuable insights, several limitations must be acknowledged. As Seladelpar is a newly emerging therapy, the limited availability of clinical studies resulted in a small sample size, potentially affecting the robustness and generalizability of our findings. Moreover, the inclusion of only three studies, primarily conducted by a single author group, raises concerns about potential publication bias. Another key limitation is the reliance on short-term follow-up data, which precludes a comprehensive evaluation of Seladelpar’s long-term efficacy and safety, particularly regarding potential adverse effects associated with prolonged use. Additionally, a lack of detailed patient-level data restricted our ability to account for pre-existing comorbidities and possible drug interactions, limiting the breadth of our therapeutic assessment. A critical gap in the included studies is the absence of immunohistochemistry-based evaluation, which hinders a deeper mechanistic understanding of Seladelpar’s biological effects on the liver, particularly its anti-fibrotic and anti-inflammatory properties. Future studies should incorporate histological and immunohistochemical analyses to elucidate its impact on hepatic fibrosis and immune modulation, with specific attention to molecular markers of liver disease progression, such as collagen deposition, inflammatory cytokines, and bile acid metabolism. Given the limited treatment options for PBC beyond UDCA and OCA, our study highlights the pressing need for alternative therapeutic strategies. To build upon our findings, future research should prioritize larger, multi-center trials with diverse patient populations, extended follow-up periods to assess long-term safety and efficacy, and mechanistic studies that integrate immunohistochemical and molecular evaluations. Furthermore, comparative studies evaluating the efficacy and safety of Seladelpar relative to other established treatments are warranted to better position its role within the current therapeutic landscape. Addressing these limitations will be crucial in defining Seladelpar’s role in PBC management and optimizing its therapeutic application.

## Conclusions

Our study findings indicate that administering Seladelpar to treat PBC results in a significant reduction in ALP levels and ALT levels and an overall improvement in the patient’s biochemical profile. We observed a favorable safety profile, with abdominal pain and headache as common complications seen in the Seladelpar group, which suggests that Seladelpar is an effective treatment option for PBC.

## Supplementary Information

Supplementary Material 1: Supplementary Table 1. Detailed search strategy used in each database. Supplementary Table 2. Risk of Bias Assessment Table. Supplementary Figure 1. Forest Plot for Biochemical Response. Supplementary Figure 2A. Forest Plot for ALP Change from baseline till longest follow-up. Supplementary Figure 2B. Forest Plot for Assessment of Heterogeneity. Supplementary Figure 3. Forest Plot for ALT Change from baseline till longest follow-up. Supplementary Figure 4. Forest Plot for Adverse Events. Supplementary Figure 5. Forest Plot for Pruritis. Supplementary Figure 6. Forest Plot for Abdominal pain. Supplementary Figure 7. Forest Plot for Headache. Supplementary Figure 8. Forest Plot for Nausea. Supplementary Figure 9. Forest Plot for total serum bilirubin. Supplementary Figure 10. Forest Plot for Any adverse event leading to treatment discontinuation. Supplementary Figure 11. Subgroup Analysis for Normalization of ALP. Supplementary Figure 12. Subgroup Analysis for Biochemical response. Supplementary Figure 13. Subgroup Analysis for ALP Change from baseline till longest follow-up. Supplementary Figure 14. Subgroup Analysis for ALT Change from baseline till longest follow-up. Supplementary Figure 15A. Regression plot for Average age. Supplementary Figure 15B. Regression plot for Female sex%. Supplementary Figure 15C. Regression plot for BMI. Supplementary Figure 15D. Regression plot for Duration of Disease.

## Data Availability

The dataset supporting the conclusions of this article are included in this article/supplementary material.
