# Correction: The role of Seladelpar in primary biliary cholangitis: a systematic review and meta-analysis

**DOI:** 10.1186/s12876-025-04214-1

**Published:** 2025-08-19

**Authors:** Taimoor Ashraf, Omar Abunada, Nandlal Seerani, Kashif Ali, Areej Muhammad, Syeda Lamiya Mir, Syed Adil Mir Shah, Muhammad Hassaan, Vikash Kumar, Waseem Abbas, Simran Bajaj, Asfa Qammar, F. N. U. Deepak, Salih Abdella Yusuf

**Affiliations:** 1 Nishtar Medical University, Multan, Pakistan; 2 Liaquat University of Medical and Health Sciences, Jamshoro, Karachi Pakistan; 3 University of Texas Rio Grande Valley, Edinburg, USA; 4 Dow Medical College, Dow University of Health Sciences, Karachi, Pakistan; 5 Jinnah Sindh Medical University, Karachi, Pakistan; 6 Shaheed Mohtarma Benazir Bhutto Medical University, Larkana, Pakistan; 7 Shaheed Mohtarma Benazir Bhutto Medical College Lyari, Karachi, Pakistan; 8 Hawassa University, Hawassa, Ethiopia

**Correction**: **BMC Gastroenterol** **25, 224 (2025)**

**https://doi.org/10.1186/s12876-025-03812-3**

Following publication of the original article an error was reported in paragraph three of the Introduction.

The final sentence originally stated: However, its use has been associated with an increase in low-density lipoprotein (LDL) levels and the need for enhanced blood pressure monitoring, though data on these effects remain limited [10].

It has been corrected to: However, its use has been associated with **a decrease** in low-density lipoprotein (LDL) levels and the need for enhanced blood pressure monitoring, though data on these effects remain limited [10].

Furthermore, reference [10] has been corrected.

Original reference 10: Study shows seladelpar beneficial for patients with primary biliary cholangitis. Available from: https://medicalxpress.com/news/2024-02-seladelpar-beneficial-patients-primary-biliary.html. Cited 2024 Jun 20.

Correct reference 10: Hirschfield GM, et al. N Engl J Med. 2024;390:783–94. doi: 10.1056/NEJMoa2312100.

The original article has been updated.

