# Supplementary material for: The role of Seladelpar in primary biliary cholangitis: a systematic review and meta-analysis
Source: BMC Gastroenterol. 2025 Apr 5;25:224. doi: 10.1186/s12876-025-03812-3 (PMC11971797; doi:10.1186/s12876-025-03812-3)
Supplement: Supplementary file 1 — Supplementary Material 1: Supplementary Table 1. Detailed search strategy used in each database. Supplementary Table 2. Risk of Bias Assessment Table. Supplementary Figure 1. Forest Plot for Biochemical Response. Supplementary Figure 2A. Forest Plot for ALP Change from baseline till longest follow-up. Supplementary Figure 2B. Forest Plot for Assessment of Heterogeneity. Supplementary Figure 3. Forest Plot for ALT Change from baseline till longest follow-up. Supplementary Figure 4. Forest Plot for Adverse Events. Supplementary Figure 5. Forest Plot for Pruritis. Supplementary Figure 6. Forest Plot for Abdominal pain. Supplementary Figure 7. Forest Plot for Headache. Supplementary Figure 8. Forest Plot for Nausea. Supplementary Figure 9. Forest Plot for total serum bilirubin. Supplementary Figure 10. Forest Plot for Any adverse event leading to treatment discontinuation. Supplementary Figure 11. Subgroup Analysis for Normalization of ALP. Supplementary Figure 12. Subgroup Analysis for Biochemical response. Supplementary Figure 13. Subgroup Analysis for ALP Change from baseline till longest follow-up. Supplementary Figure 14. Subgroup Analysis for ALT Change from baseline till longest follow-up. Supplementary Figure 15A. Regression plot for Average age. Supplementary Figure 15B. Regression plot for Female sex%. Supplementary Figure 15C. Regression plot for BMI. Supplementary Figure 15D. Regression plot for Duration of Disease. [file 12876_2025_3812_MOESM1_ESM.docx]

**Supplementary Material**

**Supplementary Table 1.** Detailed search strategy used in each database.

**Supplementary Table 2.** Risk of Bias Assessment Table

**Supplementary Figure 1.** Forest Plot for Biochemical Response

**Supplementary Figure 2A.** Forest Plot for ALP Change from baseline till longest follow-up

**Supplementary Figure 2B.** Forest Plot for Assessment of Heterogeneity

**Supplementary Figure 3.** Forest Plot for ALT Change from baseline till longest follow-up

**Supplementary Figure 4.** Forest Plot for Adverse Events

**Supplementary Figure 5.** Forest Plot for Pruritis

**Supplementary Figure 6.** Forest Plot for Abdominal pain

**Supplementary Figure 7.** Forest Plot for Headache

**Supplementary Figure 8.** Forest Plot for Nausea

**Supplementary Figure 9.** Forest Plot for total serum bilirubin

**Supplementary Figure 10.** Forest Plot for Any adverse event leading to treatment discontinuation

**Supplementary Figure 11.** Subgroup Analysis for Normalization of ALP

**Supplementary Figure 12.** Subgroup Analysis for Biochemical response

**Supplementary Figure 13.** Subgroup Analysis for ALP Change from baseline till longest follow-up

**Supplementary Figure 14.** Subgroup Analysis for ALT Change from baseline till longest follow-up

**Supplementary Figure 15A.** Regression plot for Average age

**Supplementary Figure 15B.** Regression plot for Female sex%

**Supplementary Figure 15C.** Regression plot for BMI

**Supplementary Figure 15D.** Regression plot for Duration of Disease

**Supplementary Table 1.** Detailed search strategy used in each database.

| Databases | Search string | Studies extracted |
| --- | --- | --- |
| PubMed | (Seladelpar OR peroxisome proliferator-activated receptor-delta agonist) AND (primary biliary cholangitis OR biliary cholangitis OR cholangitis OR PBC) | 59 |
| Scopus | (Seladelpar* OR peroxisome proliferator-activated receptor-delta agonist*) AND (primary biliary cholangitis* OR biliary cholangitis* OR cholangitis* OR PBC*) | 17 |
| Cochrane Library | (Seladelpar OR peroxisome proliferator-activated receptor-delta agonist) AND (primary biliary cholangitis OR biliary cholangitis OR cholangitis OR PBC) | 47 |
| Google Scholar | (Seladelpar OR peroxisome proliferator-activated receptor-delta agonist) AND (primary biliary cholangitis OR biliary cholangitis OR cholangitis OR PBC) | 207 |
| Clinical Trials.gov | Disease: Primary biliary cholangitis OR biliary cholangitis OR cholangitis OR PBC  Intervention: Seladelpar OR peroxisome proliferator-activated receptor-delta agonist | 9 |
| Science Direct | (Seladelpar OR peroxisome proliferator-activated receptor-delta agonist) AND (primary biliary cholangitis OR biliary cholangitis OR cholangitis OR PBC) | 459 |

**Supplementary Table 2.** Risk of Bias Assessment Table

|  | Cochrane Risk-of-Bias Tool | | |  |
| --- | --- | --- | --- | --- |
|  | Bias | Risk of bias | Author judgement | |
| Jones et al 2017 | Random sequence generation (selection bias) | Low Risk | Adequate randomization methods were employed, minimizing the risk of biased allocation to interventions. | |
|  | Allocation concealment (selection bias) | Low Risk | Allocation concealment was adequately described, minimizing the risk of biased allocation to interventions. | |
|  | Blinding of participants and personnel (performance bias) | Low Risk | Participants and personnel were adequately blinded to intervention allocation, minimizing performance bias. | |
|  | Blinding of outcome assessment (detection bias) | Low Risk | Outcome assessors were blinded to intervention allocation, reducing the risk of detection bias. | |
|  | Incomplete outcome data (attrition bias) | Low Risk | Incomplete outcome data were handled appropriately, minimizing the risk of attrition bias. | |
|  | Selective reporting (reporting bias) | Low Risk | There is no evidence of selective outcome reporting, reducing the risk of reporting bias. | |
|  | Other bias | Low Risk | No other biases were identified in the study that could significantly impact the results. | |
| Hirschfield et al 2023 | Random sequence generation (selection bias) | Low Risk | Adequate randomization methods were employed, minimizing the risk of biased allocation to interventions. | |
|  | Allocation concealment (selection bias) | Low Risk | Although the allocation concealment process was not explicitly described, the trial was double-blind, and patients and personnel were unaware of individual patient identification and treatment assignments. Therefore, it's likely that allocation concealment was effectively implemented, minimizing biased allocation to interventions. | |
|  | Blinding of participants and personnel (performance bias) | Low Risk | Participants and personnel were effectively blinded to intervention allocation, reducing the likelihood of performance bias. | |
|  | Blinding of outcome assessment (detection bias) | Low Risk | Outcome assessors were blinded to intervention allocation, decreasing the risk of detection bias | |
|  | Incomplete outcome data (attrition bias) | Low Risk | Incomplete outcome data were handled appropriately, minimizing the risk of attrition bias. | |
|  | Selective reporting (reporting bias) | Low Risk | There is no evidence of selective outcome reporting, reducing the risk of reporting bias. | |
|  | Other bias | Low Risk | No other biases were identified in the study that could significantly impact the results. | |
| Hirschfield et al 2024 | Random sequence generation (selection bias) | Low Risk | The study mentions random assignment in a 2:1 ratio, indicating a clear random sequence generation process | |
|  | Allocation concealment (selection bias) | Low Risk | Although the method of allocation concealment is not explicitly described, the study was double-blind, suggesting effective concealment | |
|  | Blinding of participants and personnel (performance bias) | Low Risk | The trial was described as double-blind, indicating that both participants and personnel were unaware of individual patient treatment assignments | |
|  | Blinding of outcome assessment (detection bias) | Low Risk | study's double-blind design, ensuring both participants and personnel were unaware of treatment assignments | |
|  | Incomplete outcome data (attrition bias) | Low Risk | The completion rate of the trial was high (90.2%), and methods for handling missing data are described, suggesting a low risk of bias. | |
|  | Selective reporting (reporting bias) | Low Risk | The study reports on primary and secondary endpoints as well as safety outcomes, providing comprehensive results without apparent selective reporting | |
|  | Other bias | Low Risk | The study appears to be free from other sources of bias based on the information provided. | |

**Supplementary Figure 1.** Forest Plot of Biochemical Response


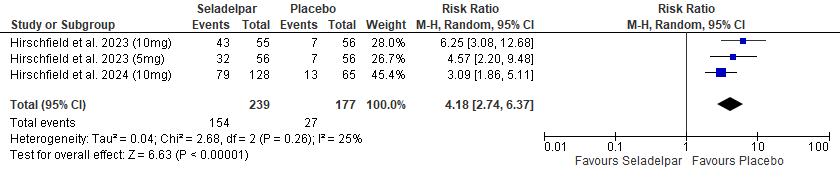


**Supplementary Figure 2A.** Forest Plot of ALP Change from baseline till longest follow-up


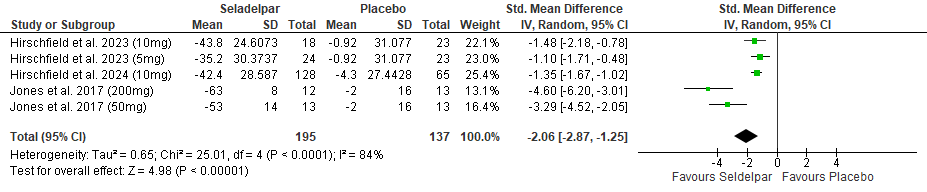


**Supplementary Figure 2B.** Forest Plot of Assessment of Heterogeneity


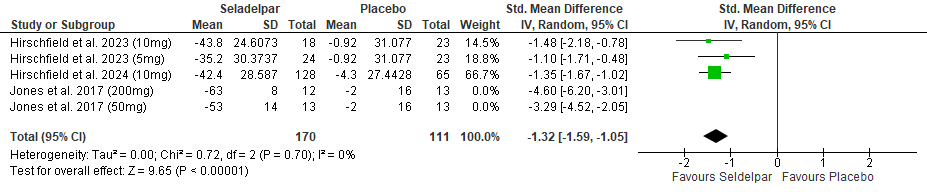


**Supplementary Figure 3.** Forest Plot ALT Change from baseline till longest follow-up


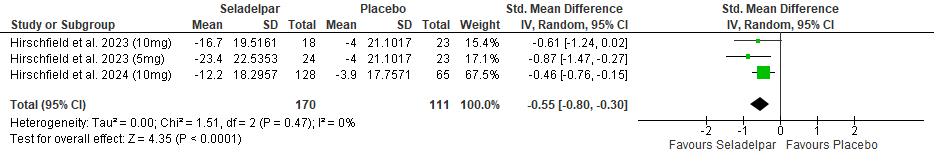


**Supplementary Figure 4.** Forest Plot of Adverse Events


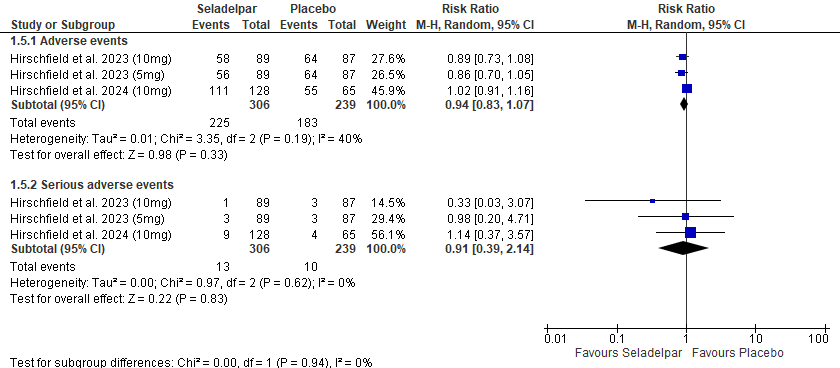


**Supplementary Figure 5.** Forest Plot of Pruritis


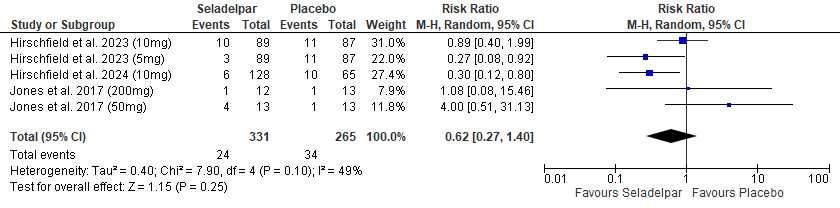


**Supplementary Figure 6.** Forest Plot of Abdominal pain


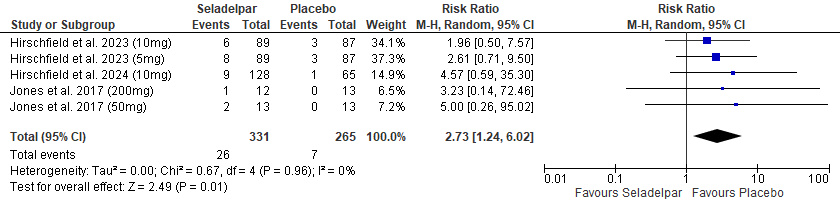


**Supplementary Figure 7.** Forest Plot of Headache


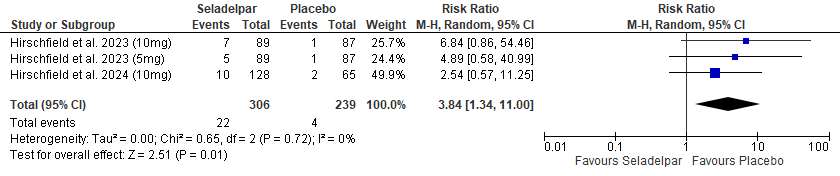


**Supplementary Figure 8.** Forest Plot of Nausea


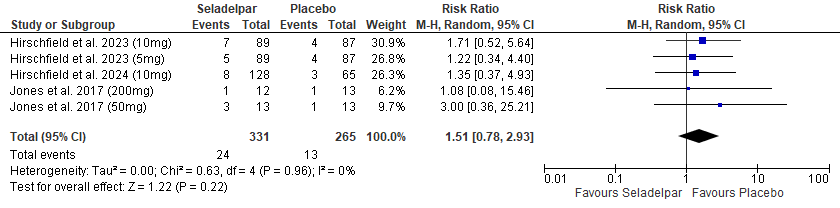


**Supplementary Figure 9.** Forest Plot of total serum bilirubin


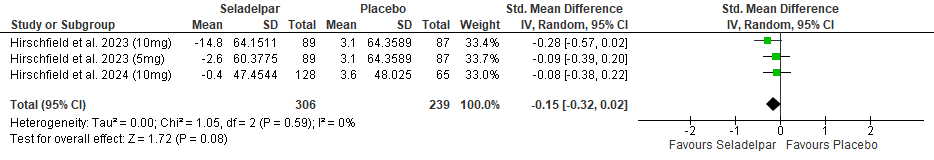


**Supplementary Figure 10.** Forest Plot of Any adverse event leading to treatment discontinuation

**
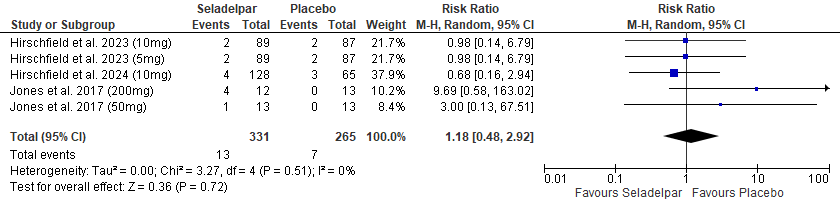
**

**Supplementary Figure 11.** Subgroup Analysis of Normalization of ALP


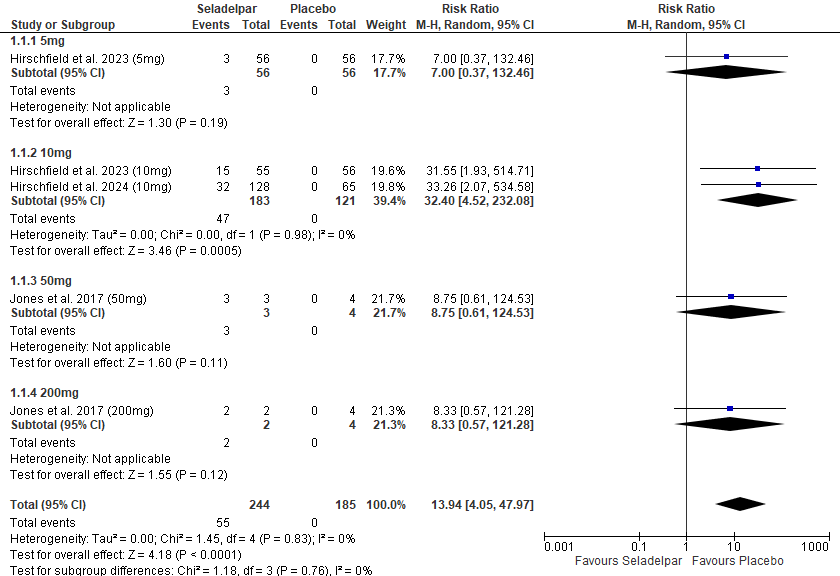


**Supplementary Figure 12.** Subgroup Analysis of Biochemical response


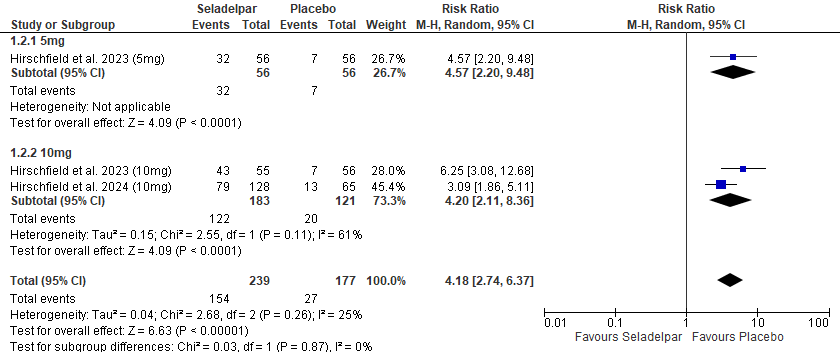


**Supplementary Figure 13.** Subgroup Analysis of ALP Change from baseline till longest follow-up


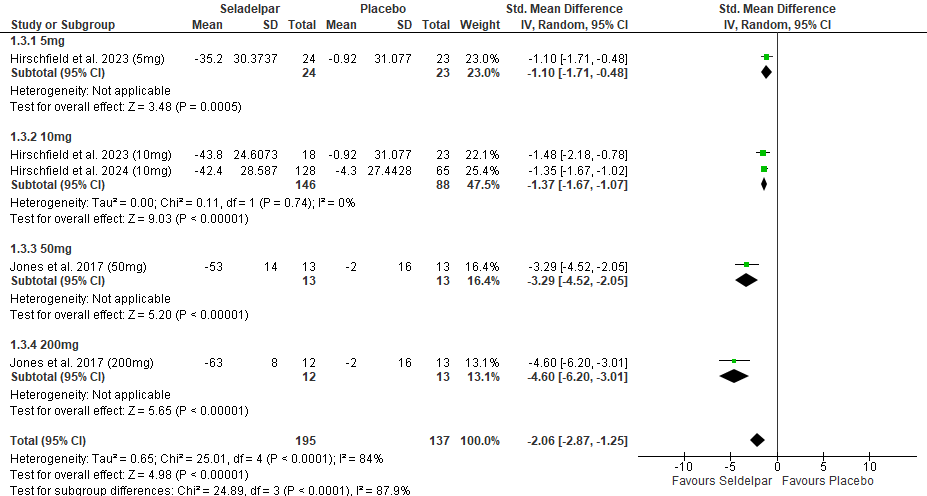


**Supplementary Figure 14.** Subgroup Analysis of ALT Change from baseline till longest follow-up


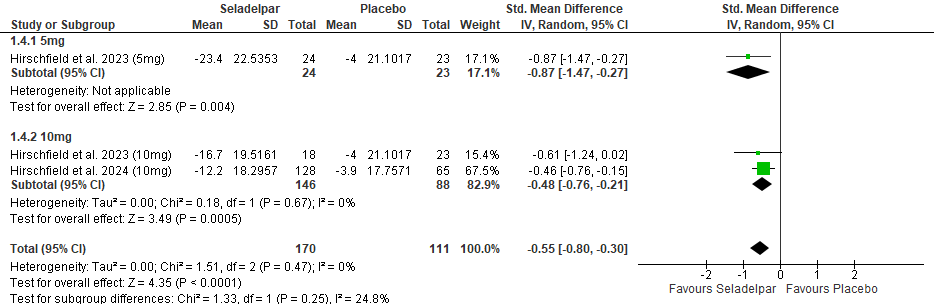


**Supplementary Figure 15A.** Regression plot for Average age

**Supplementary Figure 15B.** Regression plot for Female sex%

**Supplementary Figure 15C.** Regression plot for BMI

**Supplementary Figure 15D.** Regression plot for Duration of Disease
